# Supplementary material for: Sodium Butyrate Induces CRC Cell Ferroptosis via the CD44/SLC7A11 Pathway and Exhibits a Synergistic Therapeutic Effect with Erastin
Source: Cancers (Basel). 2023 Jan 9;15(2):423. doi: 10.3390/cancers15020423 (PMC9856855; doi:10.3390/cancers15020423)
Supplement: Supplementary file 1 [file cancers-15-00423-s001.zip › supplementary tables.pdf]

Supplementary Table S1. The values of fold change, *Padj* and Log2FC of each gene in FHC group VS HCT-116 group in PCR array.

	FHC			
ID		fold		fold Mean
CP	1.064370182	0.939522749	1.071773463	1.025222131
CISD1	1.042465761	0.965936329	0.993092495	1.000498195
LOX	0.777366405	1.122462048	1.146047362	1.015291939
GSTA1	0.037681495	5.351710219	4.9588308	3.449407505
CD44	0.957050307	0.983956654	1.061913804	1.000973588
SAT1	0.534649999	1.325619442	1.410949807	1.090406416
GSTP1	0.974904856	0.98851402	1.037659659	1.000359512
EMC2	0.961483052	1.002313162	1.037659659	1.000485291
AKR1B1	1.25411241	0.899170536	0.886791389	1.013358111
ATP5G3	1.00695555	0.965936329	1.028113827	1.000335235
ATG5	1.00695555	1.035264924	0.959264119	1.000494864
FTL	1.194715135	0.856584019	0.977159968	1.009486374
STEAP3	1.189207115	0.946057647	0.888842681	1.008035814
ALOX15	42.32192468	0.197967201	0.1193552	14.21308236
AKR1C1	1.447269237	0.825496117	0.837019613	1.036594989
FTH1	0.876605721	1.071773463	1.064370182	1.004249789
HMOX2	1.23399225	0.819793998	0.98851402	1.014100089
ATF4	0.614151575	1.186462635	1.37236731	1.057660507
USP7	0.91594529	1.023373892	1.066832243	1.002050475
STIM1	0.759611332	1.200248667	1.09682498	1.018894993
SLC1A5	0.810377861	1.076737568	1.146047362	1.011054264
GCLC	0.993092495	0.972654947	1.035264924	1.000337456
SAT2	1.025741121	0.868541486	1.122462048	1.005581552
NFE2L2	1.052144848	0.941696017	1.009284801	1.001041889
HSF1	0.946057647	1.00695555	1.049716684	1.00090996
TFR2	1.536875181	0.716977624	0.907519155	1.053790654
BRAF	0.968170696	1.023373892	1.009284801	1.000276463
KRAS	1.025741121	0.937354497	1.040059934	1.001051851
PANX2	1.208597056	1.016304932	0.814131268	1.013011085

NQO1	1.205807828	0.882702996	0.939522749	1.009344524
CS	1.109569472	0.946057647	0.952637998	1.002755039
NRAS	1.143402487	0.854607174	1.023373892	1.007127851
TXNRD1	1.052144848	0.935191248	1.016304932	1.001213676
GCLM	1.170128253	0.92444966	0.92444966	1.006342525
HRAS	1.172834949	0.888842681	0.959264119	1.006980583
GSS	0.854607174	1.044877153	1.119871604	1.006451977
MAP1LC3C	10.056107	4.0278222	0.024688791	4.702872662
ELAVL1	1.146047362	0.892959511	0.977159968	1.005388947
MAP1LC3B	0.933032992	1.01395948	1.057018041	1.001336837
RPL8	1.01161944	0.983956654	1.004631674	1.000069256
TF	1.061913804	4.552524138	0.206851406	1.940429783
NOX1	174.8531529	0.018581361	0.307786103	58.39317344
GPX4	1.044877153	0.897095409	1.066832243	1.002934935
PRNP	0.801069878	1.064370182	1.172834949	1.012758336
HSPB1	1.248330549	0.920187651	0.870550563	1.013022921
CISD2	1.079228237	0.870550563	1.064370182	1.004716327
VDAC3	1.146047362	0.899170536	0.970410231	1.005209376
PCBP1	1.127660927	0.98851402	0.897095409	1.004423452
SLC39A14	0.850667161	1.040059934	1.130269389	1.006998828
CHAC1	0.542113435	1.4913994	1.236846673	1.090119836
CARS1	0.601512518	1.334839854	1.245449622	1.060600665
CA9	0.884744831	1.044877153	1.081724666	1.003782217
NOX3	1.044877153	1.23399225	0.775572381	1.018147261
PRDX6	0.993092495	0.946057647	1.064370182	1.001173442
BBC3	0.901250463	1.049716684	1.057018041	1.002661729
EPRS	0.864537231	1.094293701	1.057018041	1.005282991
NCOA4	1.112136086	0.968170696	0.92873141	1.003012731
BECN1	1.122462048	1.01161944	0.880665874	1.004915787
SLC40A1	1.569168196	0.840896415	0.757858283	1.055974298
HARS	1.265756594	0.876605721	0.901250463	1.014537593
PPARG	0.98851402	0.961483052	1.052144848	1.000713974
SLC7A11	0.784584098	1.140763716	1.117287138	1.014211651

FSP1	0.810377861	1.154018752	1.069299999	1.011232204
DMT1	0.886791389	1.032875715	1.091768265	1.00381179
ACO1	0.911722489	0.963707118	1.138131035	1.004520214
GOT1	1.025741121	0.950439478	1.025741121	1.000640574
AKR1B10	1.356604327	0.876605721	0.840896415	1.024702155
ACSL4	0.954841604	1.016304932	1.03049202	1.000546185
ALDH1A1	2.496661098	0.378929142	1.057018041	1.310869427
TP53	1.156688184	0.870550563	0.993092495	1.006777081
KEAP1	1.205807828	0.920187651	0.901250463	1.00908198
HMOX1	1.385109468	0.840896415	0.858565436	1.02819044
IREB2	1.257013375	0.901250463	0.882702996	1.013655611
PTGES2	1.066832243	0.909618394	1.03049202	1.002314219
LPCAT3	1.104454001	0.903335201	1.002313162	1.003367454
SLC3A2	0.79922115	1.08422687	1.154018752	1.012488924
HFE	0.986232704	0.986232704	1.028113827	1.000193079
BRD4	0.856584019	1.061913804	1.099362113	1.005953312
TFR1	1.262835451	0.886791389	0.892959511	1.01419545
MAP1LC3A	7.293779908	1.954319937	0.070153878	3.106084574
CYBB	0.418509806	2.537368988	0.941696017	1.299191604
SLC39A8	1.109569472	0.920187651	0.979420298	1.00305914
GLS2	0.864537231	1.035264924	1.117287138	1.005696431

		HCT-116		
ID		fold		fold Mean
CP	0.008628547	0.00269282	0.005660683	0.005660683
CISD1	1.639587997	1.605845764	1.794190818	1.679874859
LOX	0.000357443	0.000695337	0.000367492	0.000473424
GSTA1	499.1512861	658.6340707	761.8342471	639.8732013
CD44	116.7004785	123.3545112	81.38362665	107.1462054
SAT1	142.0248924	91.77313587	128	120.5993428
GSTP1	22.89033492	14.28735218	20.06579855	19.08116188
PCBP2	5.004872558	3.490257151	4.970301378	4.488477029
EMC2	6.48302289	4.913212597	7.603515822	6.333250436

AKR1B1	0.25	0.167240944	0.216134308	0.211125084
VDAC2	4.669711215	3.080860445	4.037139205	3.929236955
ATP5G3	2.934944726	2.118926189	3.059579387	2.704483434
ATG5	6.513050141	3.899619423	6.513050141	5.641906568
FTL	5.979396995	3.580100284	5.979396995	5.179631424
STEAP3	5.749088874	3.740764991	6.696156904	5.395336923
ALOX15	327.7987782	400.7799891	615.9488185	448.1758619
AKR1C1	0.046284299	0.019192237	0.028491328	0.031322621
FTH1	2.537368988	1.870382496	2.854688508	2.420813331
HMOX2	5.52765152	2.861291865	5.121855909	4.503599764
ATF4	7.727490631	3.784230587	6.02098699	5.844236069
USP7	4.981798489	2.560927954	4.367073058	3.969933167
STIM1	6.711646198	3.196884599	5.451550708	5.120027168
SLC1A5	11.87618857	5.063026376	9.9176616	8.95229218
GCLC	10.36273802	4.510643708	8.07427841	7.649220044
SAT2	2.969047141	1.741101127	2.549121255	2.419756507
NFE2L2	4.367073058	2.183536529	3.697798641	3.416136076
HSF1	5.476800516	2.590684504	5.145578678	4.404354566
TFR2	0.156402267	0.030891063	0.173539181	0.120277504
BRAF	5.451550708	2.490899245	3.990768706	3.977739553
KRAS	6.453134074	2.808889751	4.691339797	4.651121207
PANX2	7.193364285	2.941733728	6.393769198	5.509622404
NQO1	10.95360103	4.093495568	8.713989249	7.92036195
CS	4.637455164	2.118926189	4.093495568	3.61662564
HEPH	1220.564934	1555.684008	3089.876228	1955.375057
NRAS	3.723518864	1.785919022	3.152872144	2.887436677
TXNRD1	5.683054957	2.308037504	4.778860541	4.256651
GCLM	4.438277888	2	3.317278183	3.251852024
HRAS	4.669711215	2.018569602	3.689264774	3.459181864
GSS	3.646889954	1.713168038	3.309622491	2.889893494
MAP1LC3C	64.14804236	21.45635836	66.41021819	50.67153964
ELAVL1	5.696200782	2.219138944	4.531535541	4.148958423
MAP1LC3B	7.853486842	3.167475221	4.605421921	5.208794661

RPL8	11.63178014	4.438277888	6.233316637	7.434458221
TF	35.50622311	23.10286713	68.5935016	42.40086395
NOX1	1170.844147	2903.009008	1195.446071	1756.433075
GPX4	6.805337288	2.406050072	5.121855909	4.777747756
DPP4	1215681.046	334891.2911	669782.5821	740118.3065
PRNP	8.018505295	2.776626901	5.004872558	5.266668251
HSPB1	2.032609864	1.295342252	2.178497312	1.835483143
CISD2	6.161720889	2.400497333	3.613340803	4.058519675
VDAC3	3.294364069	1.613283518	2.099433367	2.335693652
PCBP1	3.596682143	1.450617005	3.196884599	2.748061249
SLC39A14	5.169411323	1.815038311	3.60500185	3.529817161
CHAC1	16.56423878	4.112455307	10.26740718	10.31470042
CARS1	15.24220797	4.469148552	7.889861636	9.200406052
CA9	0.880665874	0.244289992	0.254663952	0.459873273
NOX3	1320.315196	901.8018546	3365.643529	1862.58686
PRDX6	1.594753377	1.081724666	1.487957514	1.388145186
BBC3	30.343873	5.630774336	18.29443979	18.08969571
EPRS	6.423383055	1.977028041	3.76678407	4.055731722
NCOA4	3.990768706	1.460706845	2.706947048	2.7194742
BECN1	9.646462622	2.531513188	5.028053498	5.735343103
SLC40A1	13.02610028	3.76678407	5.401399785	7.398094712
HARS	4.479486416	1.457335791	2.700699892	2.879174033
PPARG	9.168378896	2.422785474	4.131502861	5.240889077
SLC7A11	5.591879737	1.583737611	3.167475221	3.447697523
FSP1	0.533333333	0.923333333	0.603333333	0.686666667
DMT1	2.751083636	1.148698355	1.945309895	1.948363962
ACO1	2.789487333	1.094293701	2.143546925	2.00910932
GOT1	35.50622311	6.190259974	14.12324794	18.60657701
AKR1B10	0.733736182	0.330640037	0.73883972	0.601071979
ACSL4	3.854828474	1.211392737	2.43963728	2.50195283
ALDH1A1	8.359017223	6.980514301	1.447269237	5.595600254
TP53	2.996614154	1.119871604	1.990779358	2.035755039
KEAP1	7.378529549	1.650992233	3.189506754	4.073009512

HMOX1	17.30759466	2.271008858	5.630774336	8.403125951
IREB2	2.255321854	0.922316194	1.870382496	1.682673514
SQSTM1	4.009252647	1.016304932	2.133664486	2.386407355
PTGES2	2.260538779	0.892959511	1.620755722	1.591418004
LPCAT3	3.26405794	0.87458267	1.470866864	1.869835825
SLC3A2	1.741101127	0.812252396	1.536875181	1.363409568
HFE	1.251218139	0.319377223	0.470848009	0.680481124
BRD4	1.918528239	0.732042848	1.484523571	1.378364886
TFR1	3.073750363	0.697371833	1.319507911	1.696876702
MAP1LC3A	0.376311687	1.505246747	25.63423608	9.171931506
CYBB	1.981601227	0.001126971	5.302477767	2.428401988
SLC39A8	1.551144762	0.743978757	1.044877153	1.113333557
GLS2	0.981685855	0.03883048	1.96337171	0.994629348

ID	P	Log2FC	-LGFDR	FDR
CP	1.86515E-05	-7.500744608	2.784804082	0.00164133
CISD1	0.000397976	0.7476352	1.756690927	0.017510924
LOX	0.00104276	-11.06647447	1.707958041	0.019590339
GSTA1	0.001136358	7.53529366	1.707958041	0.019590339
CD44	0.001233437	6.742033041	1.707958041	0.019590339
SAT1	0.001335705	6.789212278	1.707958041	0.019590339
GSTP1	0.002033768	4.253558544	1.629459855	0.023471462
PCBP2	0.002275449	2.161032084	1.629459855	0.023471462
EMC2	0.00240049	2.662246172	1.629459855	0.023471462
AKR1B1	0.002836659	-2.262974197	1.602710228	0.024962597
VDAC2	0.003182562	1.973417516	1.594133097	0.025460499
ATP5G3	0.004489446	1.434869499	1.482505791	0.032922606
ATG5	0.005980428	2.495469013	1.392728406	0.040482898
FTL	0.006630189	2.359228004	1.380119424	0.041675477
STEAP3	0.007450708	2.420166158	1.37781298	0.041897395
ALOX15	0.007747714	4.978773582	1.37781298	0.041897395
AKR1C1	0.008093815	-5.048503576	1.37781298	0.041897395
FTH1	0.008843419	1.269373679	1.364169634	0.043234493

HMOX2	0.014106398	2.15087857	1.197868047	0.063406233
ATF4	0.014719532	2.466137837	1.197868047	0.063406233
USP7	0.015131033	1.986159539	1.197868047	0.063406233
STIM1	0.016731522	2.32914609	1.193405085	0.064061177
SLC1A5	0.017301506	3.146396693	1.193405085	0.064061177
GCLC	0.01747123	2.934825887	1.193405085	0.064061177
SAT2	0.018389545	1.266831793	1.188886347	0.064731199
NFE2L2	0.020215012	1.770863098	1.186813911	0.065040832
HSF1	0.020290095	2.137618417	1.186813911	0.065040832
TFR2	0.02069481	-3.13114955	1.186813911	0.065040832
BRAF	0.025282593	1.99155002	1.149426194	0.070888177
KRAS	0.025644482	2.216061835	1.149426194	0.070888177
PANX2	0.026441797	2.443303487	1.149426194	0.070888177
NQO1	0.026838272	2.97214766	1.149426194	0.070888177
CS	0.027079325	1.850675055	1.149426194	0.070888177
HEPH	0.027388614	10.43010806	1.149426194	0.070888177
NRAS	0.031773651	1.519542468	1.10517899	0.078491207
TXNRD1	0.032110039	2.087968907	1.10517899	0.078491207
GCLM	0.034004633	1.692140176	1.100595334	0.079324011
HRAS	0.034532266	1.780394999	1.100595334	0.079324011
GSS	0.035154959	1.521737988	1.100595334	0.079324011
MAP1LC3C	0.036821198	3.429561491	1.09597907	0.08017167
ELAVL1	0.03735271	2.044995466	1.09597907	0.08017167
MAP1LC3B	0.038601775	2.379022204	1.0922849	0.08085653
RPL8	0.040878749	2.894127699	1.0922849	0.08085653
TF	0.041311811	4.449645528	1.0922849	0.08085653
NOX1	0.042113784	4.910705079	1.0922849	0.08085653
GPX4	0.042265913	2.252102673	1.0922849	0.08085653
DPP4	0.044859741	19.3000505	1.07575842	0.083992707
PRNP	0.049142442	2.378600626	1.045301835	0.090094476
HSPB1	0.050816908	0.857493047	1.04383367	0.090399563
CISD2	0.051363388	2.014165381	1.04383367	0.090399563
VDAC3	0.057847077	1.21635503	1.007912322	0.098194616

PCBP1	0.058024091	1.45204654	1.007912322	0.098194616
SLC39A14	0.060427066	1.809531452	0.998561692	0.100331732
CHAC1	0.062752308	3.242143277	0.996465101	0.100817262
CARS1	0.063010789	3.116815977	0.996465101	0.100817262
CA9	0.067892128	-1.126138035	0.971885933	0.10668763
NOX3	0.07077657	10.83714575	0.961502672	0.10926909
PRDX6	0.072900147	0.471466541	0.956216919	0.110607119
BBC3	0.074774725	4.173261274	0.952614515	0.111528404
EPRS	0.077826126	2.012360541	0.942683857	0.114108013
NCOA4	0.0790976	1.438987821	0.942683857	0.114108013
BECN1	0.085873606	2.512805187	0.914049317	0.121885118
SLC40A1	0.091246833	2.80857905	0.894640079	0.127455893
HARS	0.103107974	1.504832672	0.849900123	0.141286243
PPARG	0.104359157	2.388781896	0.849900123	0.141286243
SLC7A11	0.106283094	1.765274455	0.848597074	0.141710792
FSP1	0.109833587	-0.558432477	0.841187827	0.144149179
DMT1	0.112920944	0.956774408	0.841187827	0.144149179
ACO1	0.114210925	1.000049471	0.841187827	0.144149179
GOT1	0.11466412	4.21681691	0.841187827	0.144149179
AKR1B10	0.119256994	-0.769594958	0.830291819	0.147811485
ACSL4	0.120862823	1.322266826	0.830557092	0.147721228
ALDH1A1	0.12359071	2.093768912	0.826854361	0.148986061
TP53	0.133918845	1.015819694	0.797907354	0.159254842
KEAP1	0.148407498	2.013051796	0.759122748	0.174131464
HMOX1	0.181130449	3.030818605	0.678339456	0.209729994
IREB2	0.181726551	0.7311877	0.682589669	0.207687487
SQSTM1	0.189524943	1.248931525	0.669945556	0.213823013
PTGES2	0.212846565	0.666977971	0.625077773	0.237094908
LPCAT3	0.295388427	0.898061556	0.488213839	0.32492727
SLC3A2	0.309829041	0.429312887	0.472880224	0.33660439
HFE	0.330716599	-0.555651479	0.449875187	0.354915375
BRD4	0.353015485	0.454394505	0.426801664	0.374281478
TFR1	0.398075881	0.742546032	0.379830749	0.417031876

MAP1LC3A	0.515672833	1.562128468	0.272562002	0.53387305
CYBB	0.536641084	0.902393045	0.260331861	0.549121109
SLC39A8	0.672336173	0.150479223	0.167450103	0.680064175
GLS2	0.985194292	-0.015963988	0.006478113	0.985194292

Supplementary Table S2. The values of fold change, *Padj* and Log2FC of each gene in HCT-116 group vs. HCT-116 + NaB group in PCR array.

	HCT-116			
ID	Fold			**Fold mean**
AKR1C1	1.576436072	0.653684624	0.970410231	1.066843642
SLC7A11	1.009284801	0.968170696	1.023373892	1.000276463
FSP1	0.933032992	0.993092495	1.079228237	1.001784574
HRAS	1.430645932	0.618423337	1.130269389	1.059779553
SLC40A1	2.02791896	0.586417475	0.840896415	1.151744283
CD44	1.107008782	1.170128253	0.771996743	1.016377926
NCOA4	1.591072968	0.582366793	1.079228237	1.084222666
GCLC	1.433955248	0.624165274	1.117287138	1.05846922
ACO1	1.4913994	0.585064127	1.146047362	1.074170296
ALDH1A1	1.905275996	1.591072968	0.329876978	1.275408647
CS	1.353473524	0.618423337	1.194715135	1.055537332
BECN1	1.940820463	0.509327905	1.01161944	1.153922603
NRAS	1.350349946	0.647671126	1.143402487	1.047141186
LPCAT3	2.023238881	0.542113435	0.911722489	1.159024935
TFR1	2.173469725	0.493116352	0.933032992	1.199873023
HARS	1.721102874	0.559935802	1.037659659	1.106232779
CHAC1	1.866065983	0.463294031	1.156688184	1.162016066
VDAC3	1.474269217	0.721964598	0.939522749	1.045252188
GPX4	1.554732811	0.549681057	1.170128253	1.09151404
SQSTM1	1.949809711	0.49425701	1.037659659	1.16057546
BRAF	1.440596862	0.65823136	1.05457863	1.051135617
NQO1	1.498307077	0.559935802	1.191957944	1.083400274
GOT1	2.434007027	0.424351986	0.968170696	1.275509903
STIM1	1.37236731	0.653684624	1.114708637	1.04692019
EMC2	1.040059934	0.788218036	1.21981864	1.016032203

ATG5	1.186462635	0.71038187	1.186462635	1.027769046
IREB2	1.433955248	0.586417475	1.189207115	1.069859946
HMOX2	1.277508892	0.661280073	1.183724489	1.040837818
KRAS	1.467472363	0.638754446	1.066832243	1.057686351
HSPB1	1.135504429	0.723634619	1.217003514	1.025380854
BRD4	1.501772904	0.573023681	1.162045587	1.078947391
ACSL4	1.713168038	0.538368784	1.08422687	1.111921231
HSF1	1.310393404	0.61985385	1.231144413	1.053797222
TF	0.926588062	0.602903914	1.790050142	1.106514039
GLS2	2.329467173	0.092141826	4.658934346	2.360181115
PCBP2	1.130269389	0.788218036	1.122462048	1.013649825
ELAVL1	1.477679441	0.57567774	1.175547906	1.076301696
FTL	1.186462635	0.71038187	1.186462635	1.027769046
CARS1	1.874708993	0.549681057	0.970410231	1.131600094
TP53	1.591072968	0.594603558	1.057018041	1.080898189
HFE	2.183536529	0.557354318	0.821690315	1.187527054
HEPH	0.885767519	1.128964405	2.242332156	1.41902136
GSTA1	0.791868805	1.044877153	1.208597056	1.015114338
AKR1B10	1.301341855	0.586417475	1.310393404	1.066050911
RPL8	1.697407943	0.647671126	0.909618394	1.084899154
CA9	2.318727582	0.643197335	0.670511199	1.210812038
ATP5G3	1.099362113	0.793700526	1.146047362	1.013036667
PCBP1	1.407693584	0.567752215	1.251218139	1.075554646
ALOX15	0.757858283	0.926588062	1.424050196	1.036165514
SLC3A2	1.3441244	0.627056205	1.186462635	1.052547746
MAP1LC3C	1.424050196	0.476318999	1.474269217	1.124879471
BBC3	2.075319318	0.385107556	1.251218139	1.237215004
DPP4	1.905275996	0.524858342	1.049716684	1.159950341
SLC39A14	1.598442299	0.561231024	1.114708637	1.091460653

AKR1B1	1.200248667	0.802922882	1.037659659	1.013610403
FTH1	1.064370182	0.784584098	1.197478705	1.015477662
CISD1	0.977159968	0.957050307	1.069299999	1.001170091
GSTP1	1.222640278	0.763129604	1.071773463	1.019181115
CDO1	1.356604327	1.2397077	0.594603558	1.063638528
MAP1LC3A	0.154249031	0.616996125	10.50739645	3.759547201
PPARG	2.032609864	0.537126324	0.91594529	1.161893826
PTGES2	1.522736872	0.601512518	1.091768265	1.072005885
MAP1LC3B	1.617015304	0.652176035	0.948246031	1.072479123
SAT2	1.257013375	0.737134609	1.079228237	1.02445874
USP7	1.30435207	0.670511199	1.143402487	1.039421919
STEAP3	1.09682498	0.713672127	1.277508892	1.029335333
TFR2	1.658639092	0.327598351	1.840375301	1.275537581
KEAP1	2.178497312	0.487452428	0.941696017	1.202548586
NFE2L2	1.331759279	0.66587964	1.127660927	1.041766615
PANX2	1.401203665	0.573023681	1.245449622	1.073225656
LOX	0.793700526	1.543993487	0.816014485	1.051236166
DMT1	1.501772904	0.627056205	1.061913804	1.063580971
SAT1	1.197478705	0.773782497	1.079228237	1.016829813
PRNP	1.666321368	0.577009376	1.040059934	1.094463559
SLC39A8	1.457335791	0.698984967	0.981685855	1.046002204
NOX3	0.831237896	0.567752215	2.118926189	1.172638766
VDAC2	1.231144413	0.812252396	1.064370182	1.035922331
CISD2	1.635804117	0.637280314	0.959264119	1.077449517
HMOX1	2.861291865	0.375443226	0.930879716	1.389204936
PRDX6	1.164733586	0.790041312	1.086734863	1.013836587
ATF4	1.378723669	0.675174973	1.074252648	1.042717097
CYBB	8.6938789	0.004944362	23.26356028	10.65412785
TXNRD1	1.430645932	0.581022793	1.203025036	1.071564587

GCLM	1.437272219	0.647671126	1.074252648	1.053065331
GSS	1.328685814	0.624165274	1.205807828	1.052886305
SLC1A5	1.410949807	0.601512518	1.178267139	1.063576488
EPRS	1.769489662	0.544624328	1.037659659	1.117257883
NOX1	0.733736182	1.819236788	0.749153538	1.100708836

		HCT-116 + NaB		
ID		Fold		**Fold mean**
AKR1C1	5.229476989	5.086476666	5.643799228	5.319917628
SLC7A11	0.114758002	0.12046339	0.117984289	0.117735227
FSP1	0.413702811	0.481853559	0.468677248	0.45474454
HRAS	3.801757911	3.963202453	4.218314518	3.994424961
SLC40A1	15.24220797	11.3137085	15.45498126	14.00363258
CD44	0.068789555	0.106456307	0.05784489	0.077696917
NCOA4	4.723970646	3.784230587	3.732131966	4.080111066
GCLC	2.4794154	2.428389769	2.602683711	2.503496293
ACO1	4.680513013	7.498835972	6.900336256	6.35989508
ALDH1A1	11.71268557	7.260153243	8.633825892	9.202221567
CS	2.340256506	2.921413689	3.26405794	2.841909379
BECN1	3.450168128	4.981798489	5.193354353	4.541773657
NRAS	2.776626901	4.417816003	4.448544343	3.880995749
LPCAT3	4.102964486	6.619244982	7.143676088	5.955295185
TFR1	18.50701094	37.79176517	38.8542363	31.7176708
HARS	5.290240584	10.22007114	9.80374754	8.438019754
CHAC1	3.630076621	6.320330495	6.727171322	5.559192813
VDAC3	3.160165247	2.250116969	2.29739671	2.569226309
GPX4	2.183536529	3.045473744	2.688248799	2.639086358
SQSTM1	3.145595872	5.749088874	5.553253802	4.815979516
BRAF	1.810849523	2.080119868	1.967913307	1.952960899

NQO1	0.075015542	0.033803896	0.037768658	0.048862698
GOT1	3.466148183	5.910717762	6.161720889	5.179528945
STIM1	2.422785474	5.051341805	4.07462324	3.849583506
EMC2	3.21912069	1.914100614	2.109157259	2.414126188
ATG5	2.901234011	1.887748625	1.823444977	2.204142538
IREB2	2.042024251	4.407620464	3.458148925	3.30259788
HMOX2	1.896492062	3.84593221	4.417816003	3.386746758
KRAS	1.662475792	3.017457254	2.874544437	2.518159161
HSPB1	0.36941986	0.587773953	0.604298528	0.520497447
BRD4	1.666321368	3.21912069	3.596682143	2.827374733
ACSL4	2.632925439	2.109157259	1.874708993	2.20559723
HSF1	1.433955248	2.657371628	3.010493495	2.367273457
TF	2.969047141	12.90626815	14.6213032	10.1655395
GLS2	8.51496146	50.91433496	50.91433496	36.78121046
PCBP2	0.868541486	0.242602558	0.22635619	0.445833411
ELAVL1	1.477679441	4.009252647	3.872682784	3.119871624
FTL	0.509327905	0.653684624	0.715322966	0.626111832
CARS1	1.643380629	2.901234011	3.109465621	2.551360087
TP53	0.241484082	0.486327474	0.432268616	0.386693391
HFE	0.112396317	0.052799448	0.127331976	0.097509247
HEPH	1.796264746	6.797479993	6.430807926	5.008184222
GSTA1	3.279175994	1.844632387	1.498307077	2.207371819
AKR1B10	1.866065983	21.40684088	23.26356028	15.51215571
RPL8	2.351095813	1.674039226	1.662475792	1.895870277
CA9	0.093644192	0.110593104	0.05018268	0.084806659
ATP5G3	4.397448454	1.927414237	1.785919022	2.703593904
PCBP1	1.112136086	3.899619423	3.819366416	2.943707308
ALOX15	1.057018041	6.91629785	8.51496146	5.49609245
SLC3A2	0.977159968	6.133313379	6.305744288	4.472072545

MAP1LC3C	1.2397077	11.3137085	15.56247916	9.371965119
BBC3	1.143402487	9.469795909	8.713989249	6.442395882
DPP4	0.570381858	0.226879789	0.295248165	0.364169937
SLC39A14	1.069299999	3.286761258	3.596682143	2.650914466
AKR1B1	4.150638637	1.757266904	1.639587997	2.515831179
FTH1	0.993092495	0.38958229	0.423372656	0.602015814
CISD1	3.621699045	1.543993487	1.554732811	2.240141781
GSTP1	2.02791896	1.180992661	1.337927555	1.515613059
CDO1	2.808889751	1.986184991	1.101905116	1.965659953
MAP1LC3A	0.182166974	37.35768972	33.20510909	23.58165526
PPARG	0.442372416	0.533416121	0.436282144	0.470690227
PTGES2	0.970410231	2.456606299	2.153475136	1.860163889
MAP1LC3B	4.479486416	1.561933827	1.662475792	2.567965345
SAT2	1.777685362	1.125058485	1.164733586	1.355825811
USP7	0.791868805	0.814131268	0.808507652	0.804835908
STEAP3	0.439316726	2.30271096	2.874544437	1.872190708
TFR2	0.52850902	10.55606329	1.647182035	4.243918114
KEAP1	0.254076233	2.934944726	2.854688508	2.014569823
NFE2L2	1.159363791	0.718636109	0.66587964	0.847959847
PANX2	0.735433432	1.51221856	1.823444977	1.357032323
LOX	0.380684218	0.740548776	5.339359417	2.153530804
DMT1	0.686183655	0.963707118	1.025741121	0.891877298
SAT1	2.070529848	0.823591017	0.817902059	1.237340975
PRNP	1.773582778	1.130269389	0.963707118	1.289186429
SLC39A8	1.467472363	0.604298528	0.552227	0.874665964
NOX3	0.140957616	1.733074092	0.728667896	0.867566534
VDAC2	2.37841423	0.747424624	0.742261785	1.28936688
CISD2	2.37841423	0.858565436	0.784584098	1.340521255
HMOX1	1.05457863	2.214017563	1.887748625	1.718781606

PRDX6	1.526259209	0.562529242	0.554784736	0.881191062
ATF4	1.407693584	0.684600064	0.675174973	0.922489541
CYBB	0.046070913	23.42537114	14.52030649	12.66391618
TXNRD1	0.6720622	1.21981864	1.194715135	1.028865325
GCLM	1.38831345	0.860551437	0.802922882	1.01726259
GSS	0.784584098	1.222640278	1.180992661	1.062739012
SLC1A5	0.686183655	1.271619166	1.211392737	1.05639852
EPRS	1.572797936	0.872564288	0.890898718	1.112086981
NOX1	1.757266904	1.529789694	0.000109509	1.095722036

ID	**P**	**FC**	FDR	log2FC
AKR1C1	0.000181032	4.986595426	0.00848068	2.318055159
SLC7A11	7.56889E-07	0.117702687	6.66063E-05	-3.086780845
FSP1	0.0003188	0.45393446	0.00848068	-1.139444082
HRAS	0.000385485	3.769109293	0.00848068	1.914223629
SLC40A1	0.000820813	12.15862999	0.0144463	3.603908772
CD44	0.001653317	0.076444908	0.024248645	-3.709435775
NCOA4	0.00231677	3.763167101	0.029125107	1.911947353
GCLC	0.003905279	2.365204623	0.040561217	1.241965002
ACO1	0.004148306	5.920751209	0.040561217	2.565780233
ALDH1A1	0.004817196	7.215116181	0.042391329	2.851022626
CS	0.006970149	2.692381683	0.055761189	1.428882947
BECN1	0.008026985	3.935943057	0.057623431	1.976709349
NRAS	0.008643519	3.706277434	0.057623431	1.889970879
LPCAT3	0.009897624	5.138194189	0.057623431	2.361261416
TFR1	0.010016506	26.43418945	0.057623431	4.724333185
HARS	0.010476987	7.627707222	0.057623431	2.931249469
CHAC1	0.013951028	4.784092902	0.071985647	2.258245405
VDAC3	0.014724337	2.457996585	0.071985647	1.297482911

GPX4	0.015871109	2.417821723	0.073508294	1.273707872
SQSTM1	0.01762623	4.149647895	0.077555411	2.052988926
BRAF	0.019541483	1.85795331	0.078102071	0.893714248
NQO1	0.020113118	0.045101243	0.078102071	-4.47068901
GOT1	0.020413041	4.060751651	0.078102071	2.021746797
STIM1	0.024368917	3.677055369	0.089352696	1.878550901
EMC2	0.030282065	2.376033141	0.106592868	1.248554959
ATG5	0.03736241	2.144589337	0.121593794	1.100701415
IREB2	0.038019439	3.08694413	0.121593794	1.626179372
HMOX2	0.040691023	3.253865972	0.121593794	1.702154827
KRAS	0.041192542	2.380818434	0.121593794	1.251457602
HSPB1	0.04145243	0.507613776	0.121593794	-0.978196872
BRD4	0.054664075	2.620493602	0.150484011	1.389838586
ACSL4	0.054721459	1.983591256	0.150484011	0.988114771
HSF1	0.0666783	2.246422183	0.175619229	1.167629087
TF	0.068040149	9.186995499	0.175619229	3.199593122
GLS2	0.072374102	15.58406269	0.175619229	3.961999481
PCBP2	0.076823549	0.439829812	0.175619229	-1.184982701
ELAVL1	0.077156182	2.89869619	0.175619229	1.535404133
FTL	0.077469559	0.609195066	0.175619229	-0.715023839
CARS1	0.077831249	2.254648176	0.175619229	1.172902327
TP53	0.079829817	0.357751909	0.175625598	-1.482968629
HFE	0.096746557	0.08211118	0.203327858	-3.606277529
HEPH	0.097042841	3.529322646	0.203327858	1.819391326
GSTA1	0.099657802	2.174505606	0.203950851	1.120687428
AKR1B10	0.102553527	14.55104587	0.205107054	3.863050947
RPL8	0.105459906	1.7475083	0.206232706	0.805299308
CA9	0.112036745	0.070041143	0.212771788	-3.835653569
ATP5G3	0.119200162	2.668801626	0.212771788	1.416192073

PCBP1	0.121138293	2.736920266	0.212771788	1.452553406
ALOX15	0.121595042	5.304261123	0.212771788	2.407151799
SLC3A2	0.124221525	4.248807297	0.212771788	2.087057912
MAP1LC3C	0.124925255	8.331528278	0.212771788	3.058581158
BBC3	0.126425983	5.20717568	0.212771788	2.380501081
DPP4	0.128146645	0.313953041	0.212771788	-1.671379306
SLC39A14	0.140605047	2.428776941	0.228061748	1.280229999
AKR1B1	0.143170645	2.482049487	0.228061748	1.31153188
FTH1	0.147317491	0.592840036	0.228061748	-0.754285214
CISD1	0.147721814	2.237523674	0.228061748	1.161902947
GSTP1	0.165656286	1.487089033	0.251340571	0.572491025
CDO1	0.174400995	1.848052605	0.260123517	0.886005824
MAP1LC3A	0.180553518	6.272472189	0.264811827	2.649034169
PPARG	0.19932346	0.405106057	0.287548598	-1.303628438
PTGES2	0.208204092	1.735217983	0.290996507	0.795116909
MAP1LC3B	0.208327045	2.39441989	0.290996507	1.259676168
SAT2	0.272384947	1.323455751	0.374529302	0.40430996
USP7	0.285253861	0.77431108	0.386189842	-0.369014808
STEAP3	0.326103914	1.818834589	0.434805218	0.863014346
TFR2	0.407202957	3.327160388	0.534833734	1.734291414
KEAP1	0.468608697	1.675250253	0.606434785	0.744376625
NFE2L2	0.483987284	0.813963352	0.617259145	-0.296964254
PANX2	0.52821065	1.264442678	0.664036246	0.338501636
LOX	0.532429914	2.048569935	0.659913133	1.034617145
DMT1	0.563837873	0.838560789	0.689135178	-0.254012723
SAT1	0.639102498	1.216861425	0.769398106	0.283164884
PRNP	0.65245247	1.177916266	0.769398106	0.236236986
SLC39A8	0.667541611	0.836198968	0.769398106	-0.258081832
NOX3	0.671416391	0.739841253	0.769398106	-0.434712348

VDAC2	0.673223343	1.244655938	0.769398106	0.315746991
CISD2	0.682201713	1.244161544	0.769663471	0.31517382
HMOX1	0.711137691	1.237241218	0.786130241	0.307126801
PRDX6	0.717991223	0.869164788	0.786130241	-0.202298366
ATF4	0.723597154	0.884697818	0.786130241	-0.17674333
CYBB	0.84464398	1.188639404	0.906447198	0.249311113
TXNRD1	0.897236833	0.960152414	0.951287245	-0.058664659
GCLM	0.90911903	0.966001406	0.952410413	-0.049902806
GSS	0.971395389	1.009357807	0.994319502	0.013437686
SLC1A5	0.982296946	0.993251103	0.994319502	-0.009769605
EPRS	0.990851711	0.995371791	0.994319502	-0.006692593
NOX1	0.994319502	0.995469465	0.994319502	-0.006551032
