# Supplementary figures and images for: Sodium Butyrate Induces CRC Cell Ferroptosis via the CD44/SLC7A11 Pathway and Exhibits a Synergistic Therapeutic Effect with Erastin
Source: Cancers (Basel). 2023 Jan 9;15(2):423. doi: 10.3390/cancers15020423 (PMC9856855; doi:10.3390/cancers15020423)

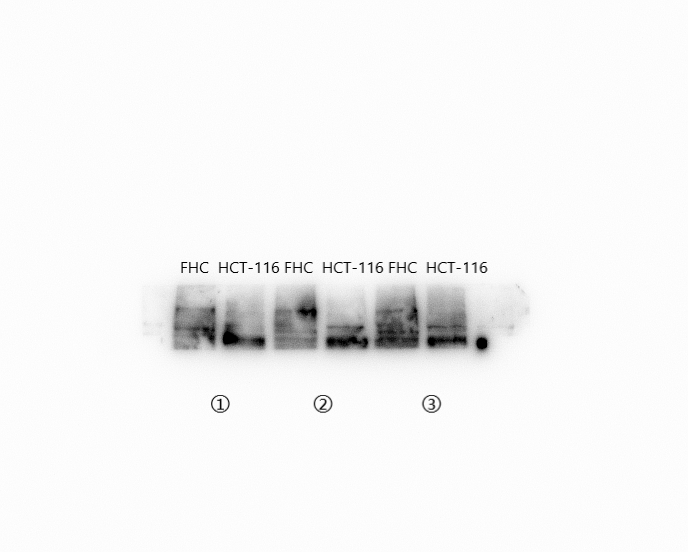

Supplement: Supplementary file 1 [file cancers-15-00423-s001.zip › supplementary figures/Figure S1. The original Western blotting figures of Figure 4A/cd44.png]

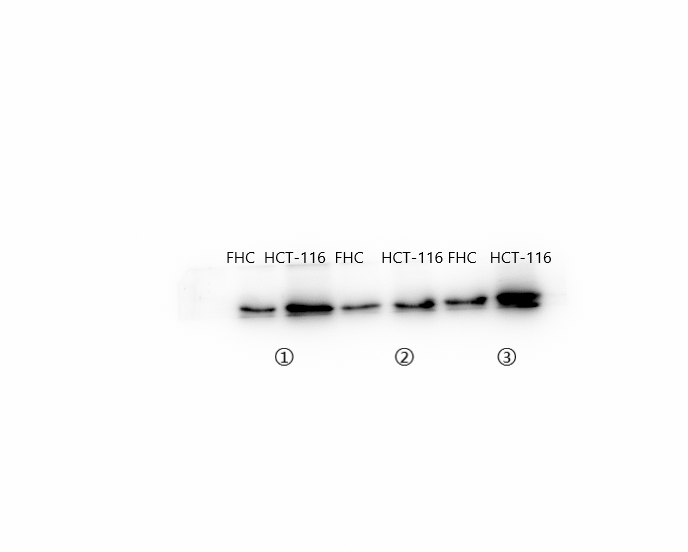

Supplement: Supplementary file 1 [file cancers-15-00423-s001.zip › supplementary figures/Figure S1. The original Western blotting figures of Figure 4A/gapdh.png]

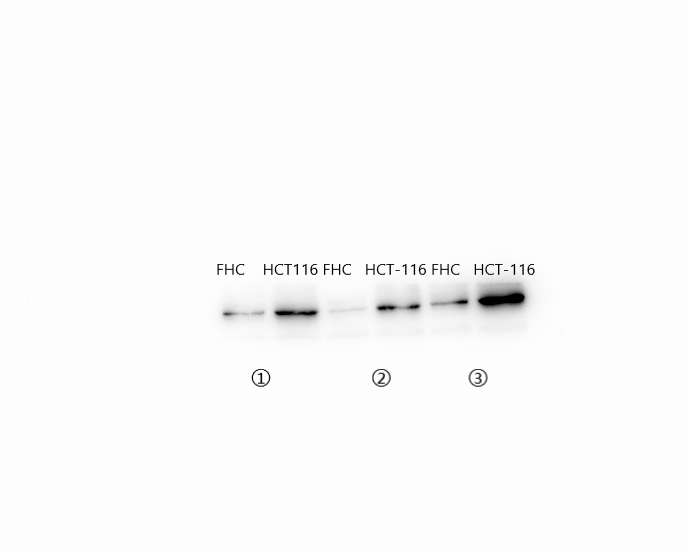

Supplement: Supplementary file 1 [file cancers-15-00423-s001.zip › supplementary figures/Figure S1. The original Western blotting figures of Figure 4A/slc7a11.png]

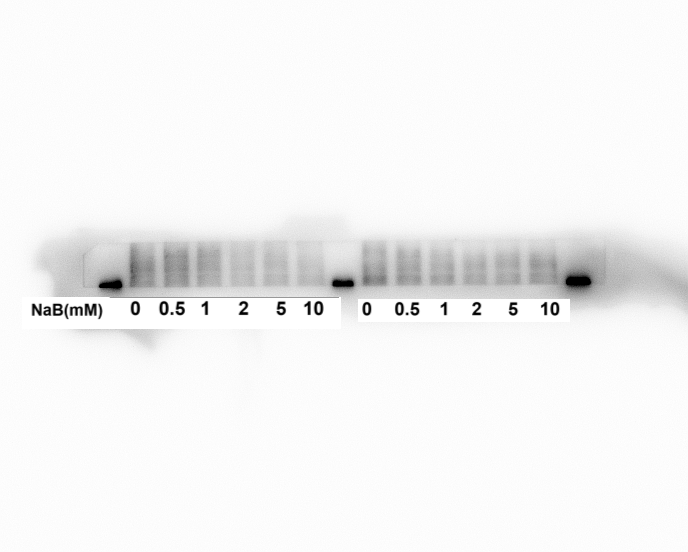

Supplement: Supplementary file 1 [file cancers-15-00423-s001.zip › supplementary figures/Figure S2. The original Western blotting figures of Figure 4B/fhc/cd44/1-2.png]

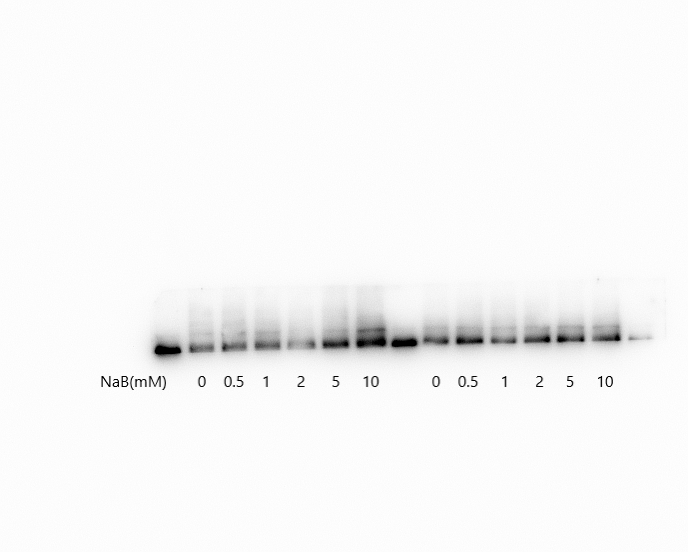

Supplement: Supplementary file 1 [file cancers-15-00423-s001.zip › supplementary figures/Figure S2. The original Western blotting figures of Figure 4B/fhc/cd44/3-4.png]

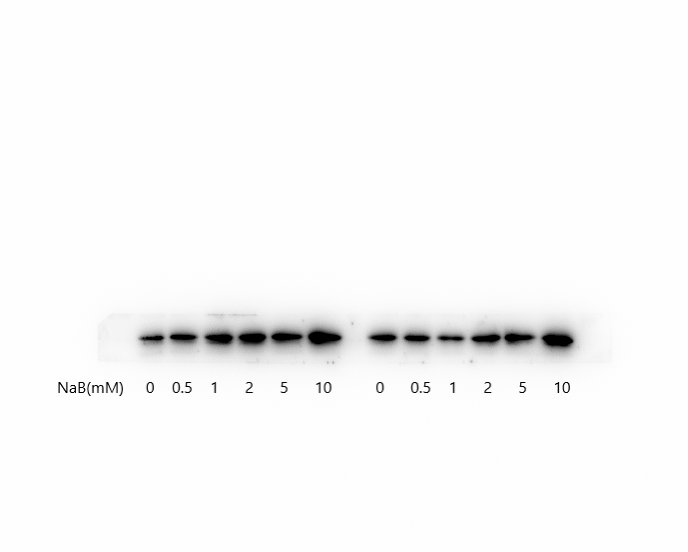

Supplement: Supplementary file 1 [file cancers-15-00423-s001.zip › supplementary figures/Figure S2. The original Western blotting figures of Figure 4B/fhc/gapdh/1-2.png]

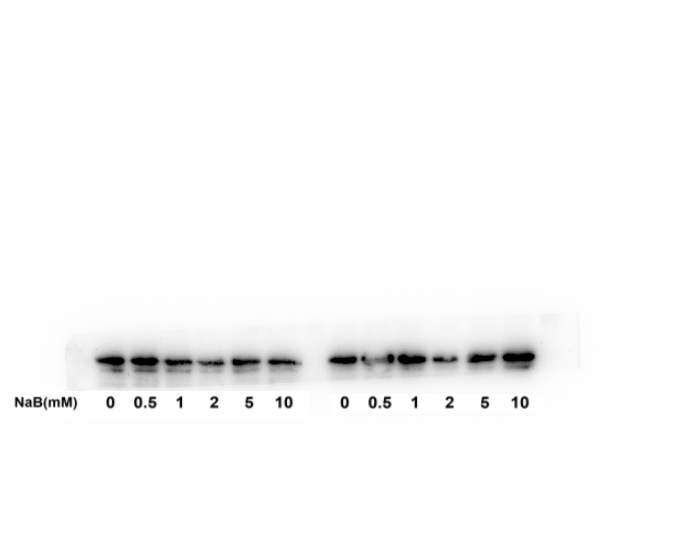

Supplement: Supplementary file 1 [file cancers-15-00423-s001.zip › supplementary figures/Figure S2. The original Western blotting figures of Figure 4B/fhc/gapdh/3-4.png]

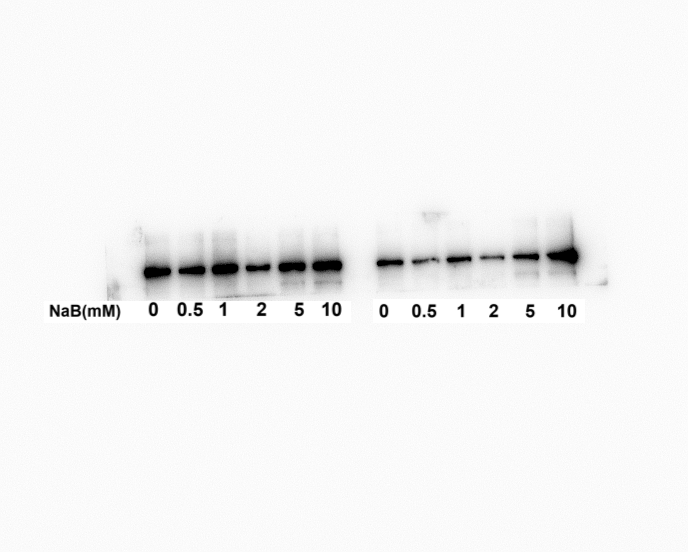

Supplement: Supplementary file 1 [file cancers-15-00423-s001.zip › supplementary figures/Figure S2. The original Western blotting figures of Figure 4B/fhc/gpx4/1-2.png]

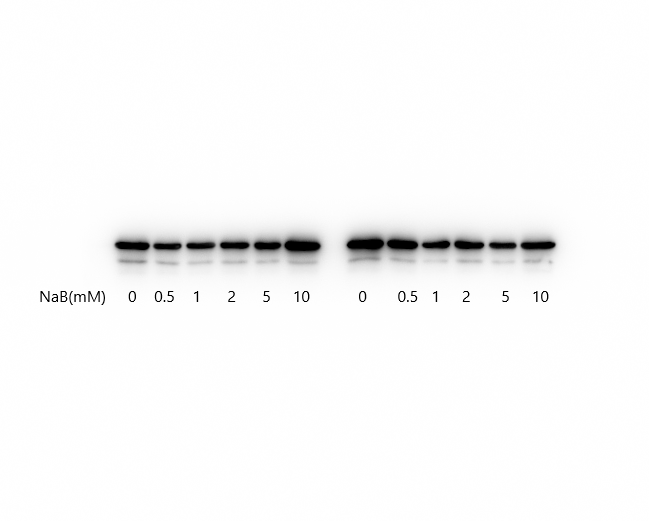

Supplement: Supplementary file 1 [file cancers-15-00423-s001.zip › supplementary figures/Figure S2. The original Western blotting figures of Figure 4B/fhc/gpx4/3-4.png]

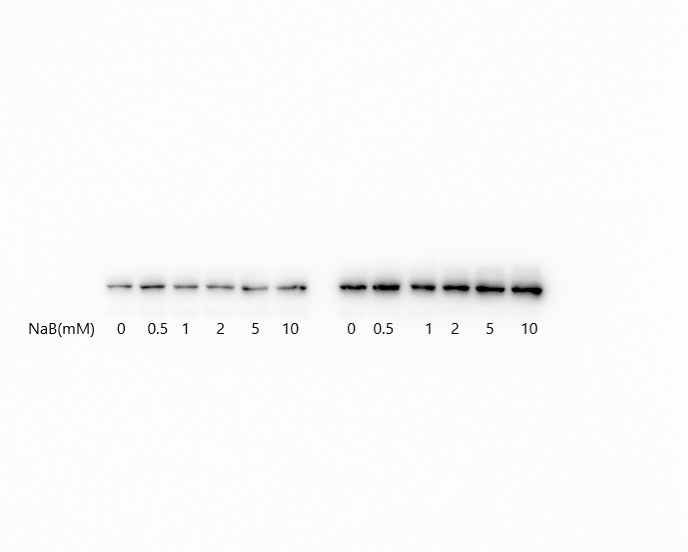

Supplement: Supplementary file 1 [file cancers-15-00423-s001.zip › supplementary figures/Figure S2. The original Western blotting figures of Figure 4B/fhc/slc7a11/1-2.png]

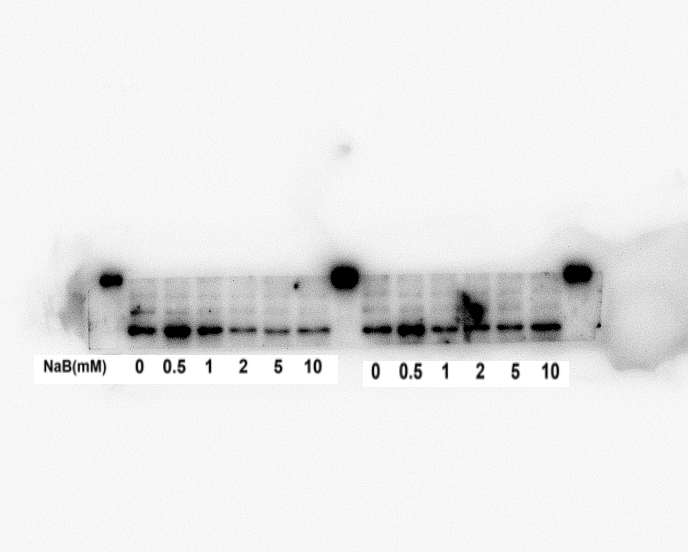

Supplement: Supplementary file 1 [file cancers-15-00423-s001.zip › supplementary figures/Figure S2. The original Western blotting figures of Figure 4B/fhc/slc7a11/3-4.png]

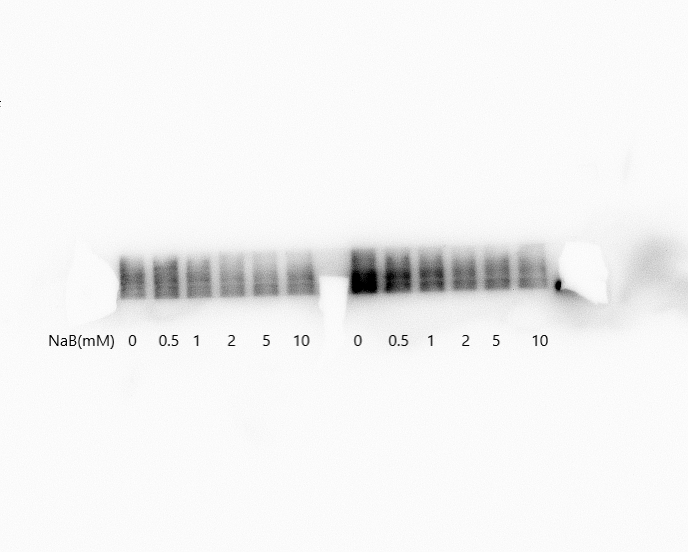

Supplement: Supplementary file 1 [file cancers-15-00423-s001.zip › supplementary figures/Figure S2. The original Western blotting figures of Figure 4B/hct-116/cd44/1-2.png]

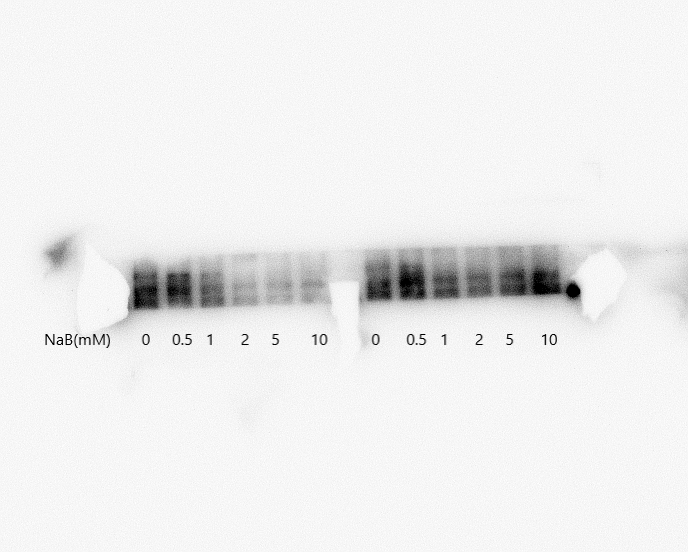

Supplement: Supplementary file 1 [file cancers-15-00423-s001.zip › supplementary figures/Figure S2. The original Western blotting figures of Figure 4B/hct-116/cd44/3-4.png]

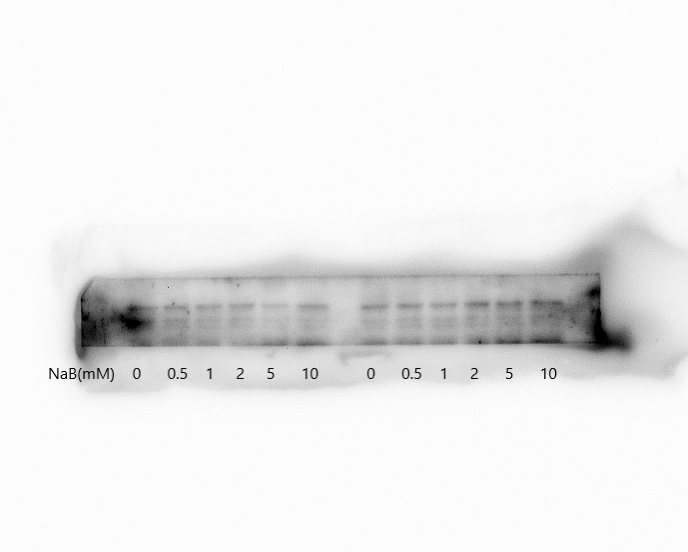

Supplement: Supplementary file 1 [file cancers-15-00423-s001.zip › supplementary figures/Figure S2. The original Western blotting figures of Figure 4B/hct-116/gapdh/1-2.png]

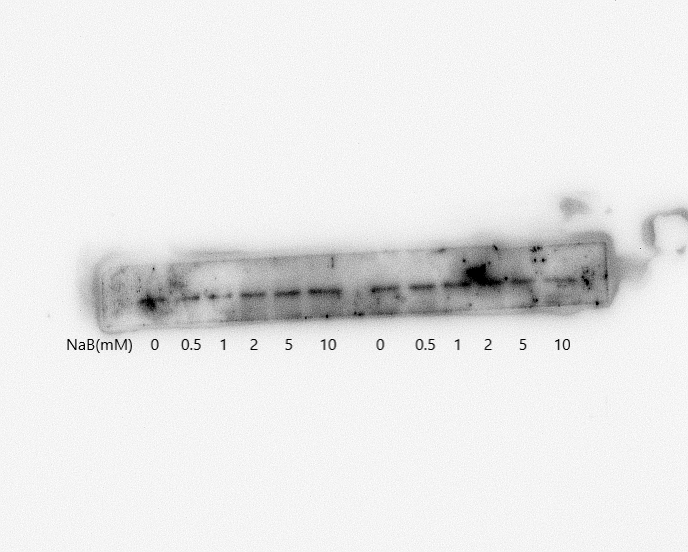

Supplement: Supplementary file 1 [file cancers-15-00423-s001.zip › supplementary figures/Figure S2. The original Western blotting figures of Figure 4B/hct-116/gapdh/3-4.png]

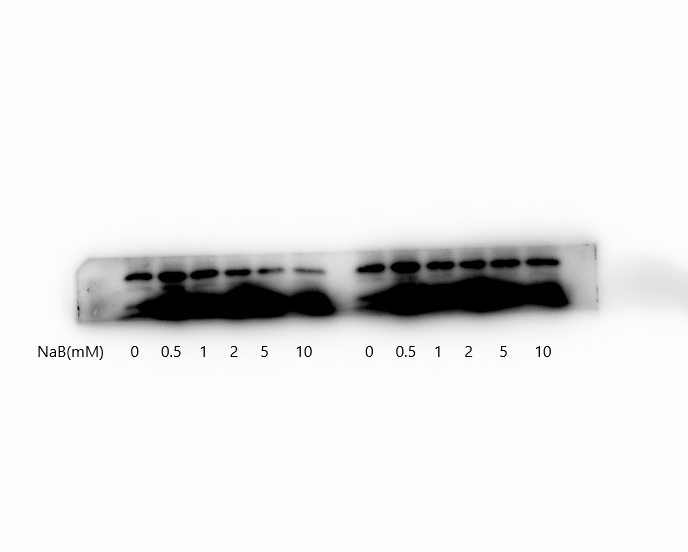

Supplement: Supplementary file 1 [file cancers-15-00423-s001.zip › supplementary figures/Figure S2. The original Western blotting figures of Figure 4B/hct-116/gpx4/1-2.png]

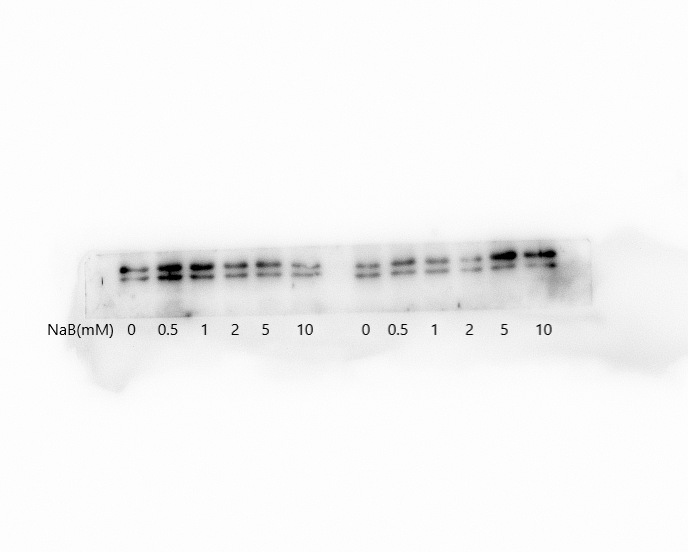

Supplement: Supplementary file 1 [file cancers-15-00423-s001.zip › supplementary figures/Figure S2. The original Western blotting figures of Figure 4B/hct-116/gpx4/3-4.png]

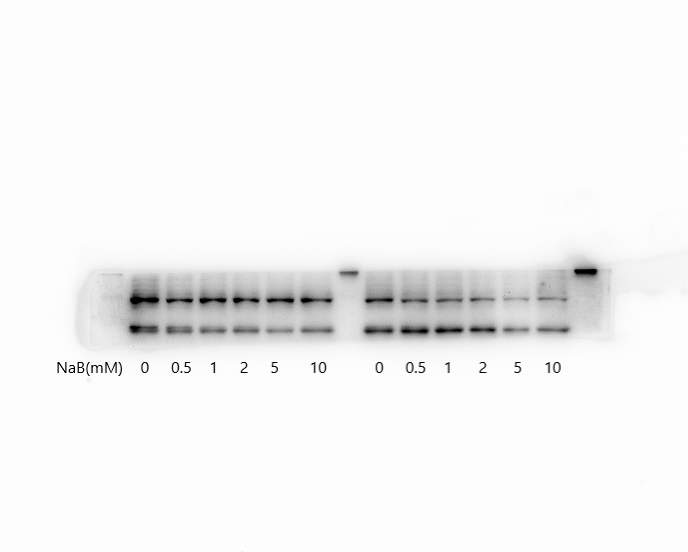

Supplement: Supplementary file 1 [file cancers-15-00423-s001.zip › supplementary figures/Figure S2. The original Western blotting figures of Figure 4B/hct-116/slc7a11/1-2.png]

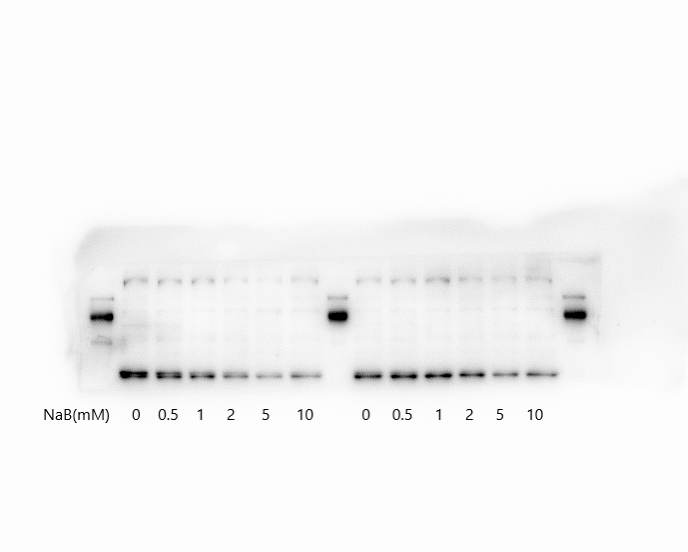

Supplement: Supplementary file 1 [file cancers-15-00423-s001.zip › supplementary figures/Figure S2. The original Western blotting figures of Figure 4B/hct-116/slc7a11/3-4.png]

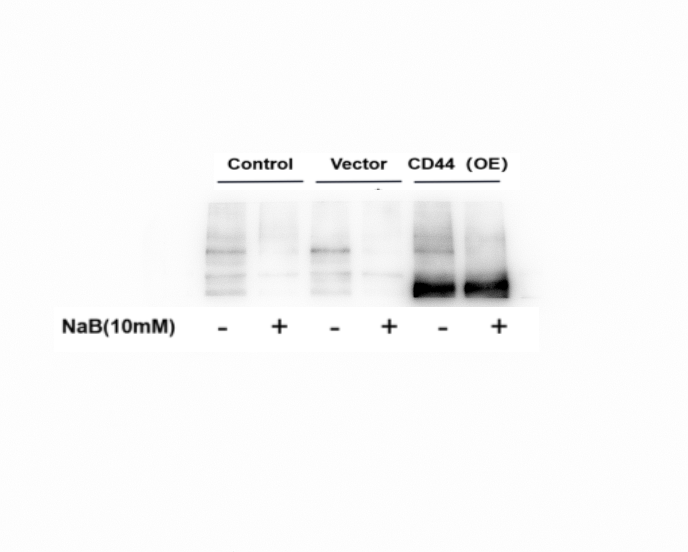

Supplement: Supplementary file 1 [file cancers-15-00423-s001.zip › supplementary figures/Figure S3. The original Western blotting figures of Figure 5B/cd44/1.png]

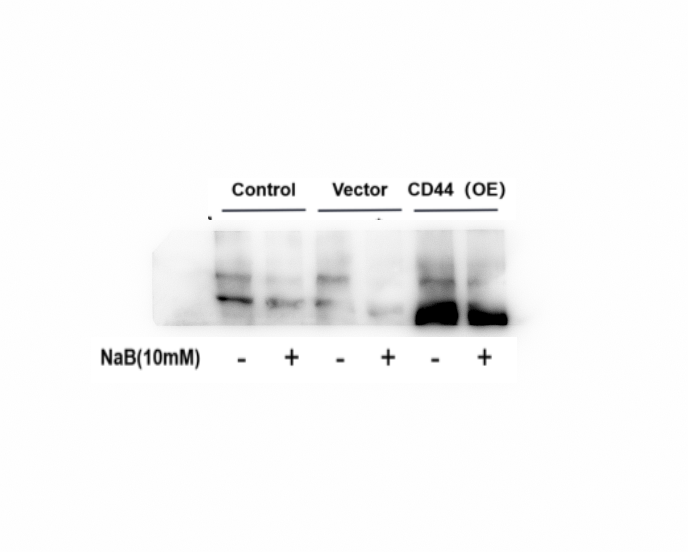

Supplement: Supplementary file 1 [file cancers-15-00423-s001.zip › supplementary figures/Figure S3. The original Western blotting figures of Figure 5B/cd44/2.png]

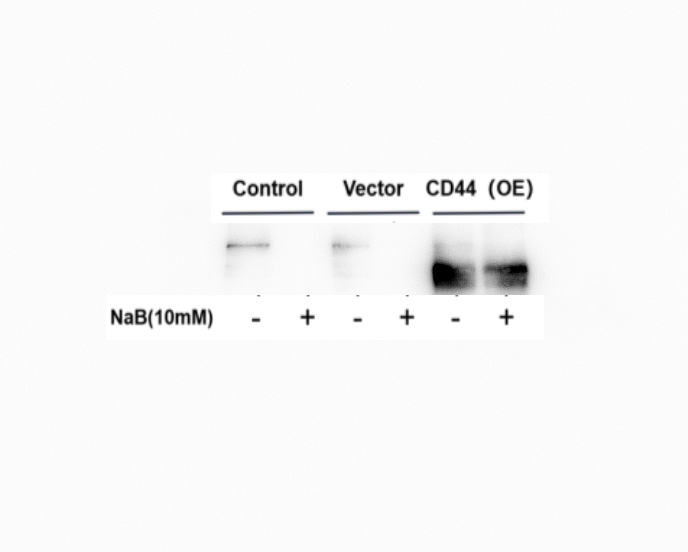

Supplement: Supplementary file 1 [file cancers-15-00423-s001.zip › supplementary figures/Figure S3. The original Western blotting figures of Figure 5B/cd44/3.png]

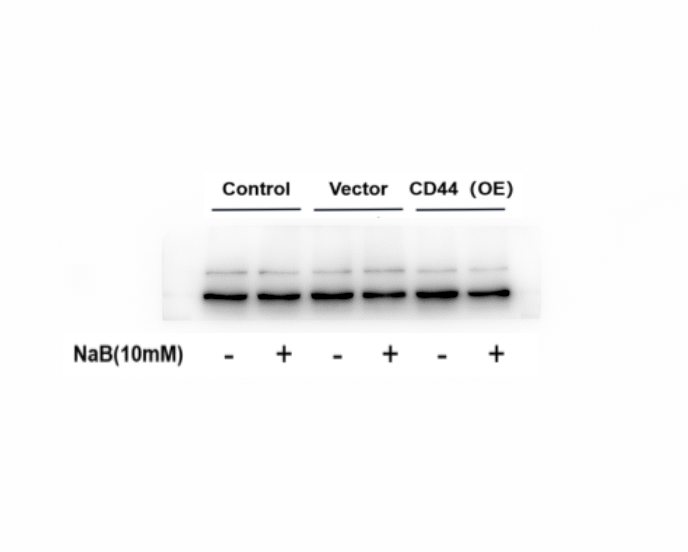

Supplement: Supplementary file 1 [file cancers-15-00423-s001.zip › supplementary figures/Figure S3. The original Western blotting figures of Figure 5B/gapdh/1.png]

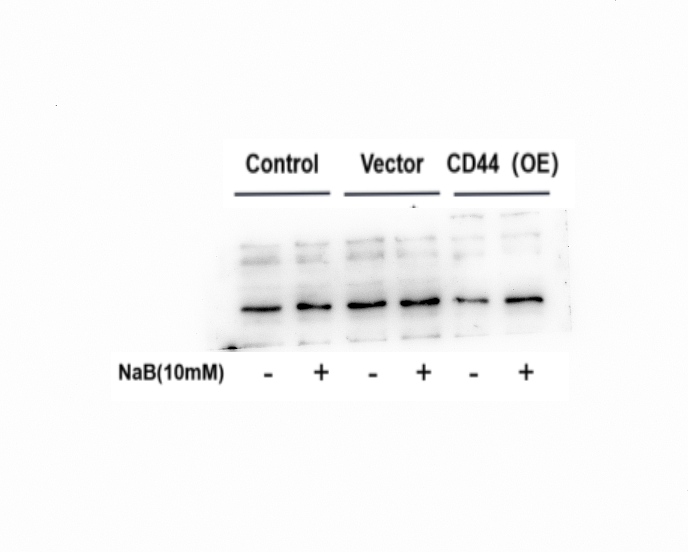

Supplement: Supplementary file 1 [file cancers-15-00423-s001.zip › supplementary figures/Figure S3. The original Western blotting figures of Figure 5B/gapdh/2.png]

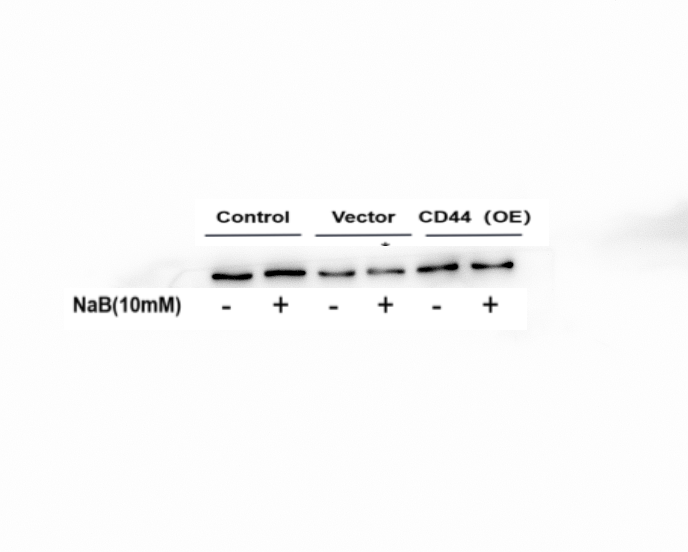

Supplement: Supplementary file 1 [file cancers-15-00423-s001.zip › supplementary figures/Figure S3. The original Western blotting figures of Figure 5B/gapdh/3.png]

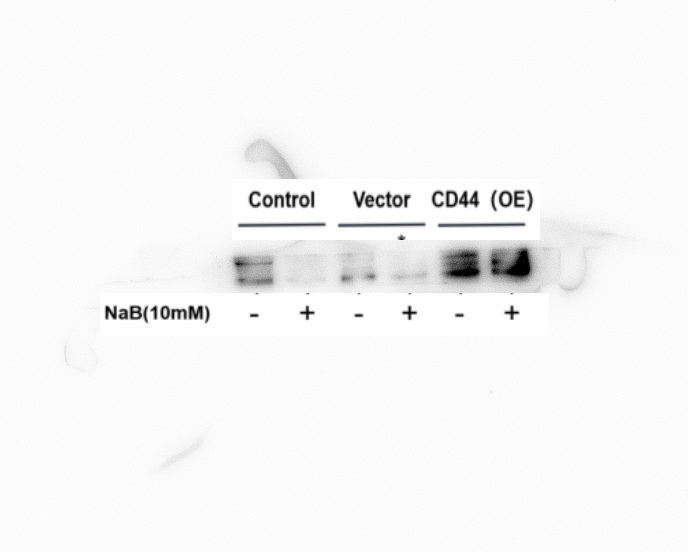

Supplement: Supplementary file 1 [file cancers-15-00423-s001.zip › supplementary figures/Figure S3. The original Western blotting figures of Figure 5B/slc7a11/1.png]

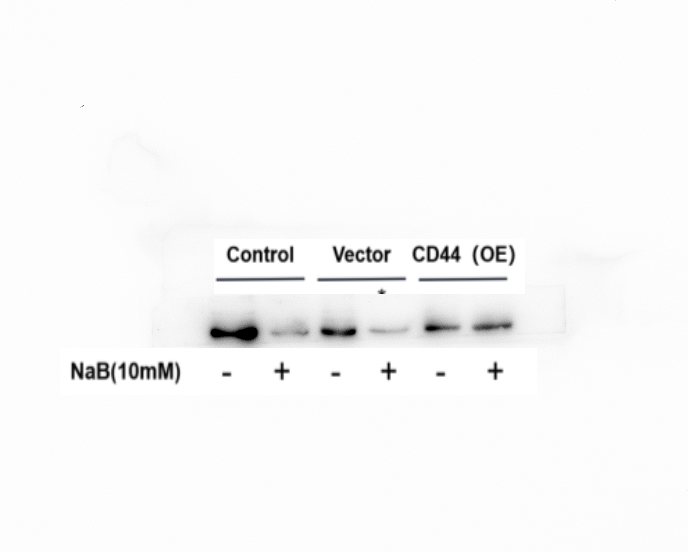

Supplement: Supplementary file 1 [file cancers-15-00423-s001.zip › supplementary figures/Figure S3. The original Western blotting figures of Figure 5B/slc7a11/2.png]

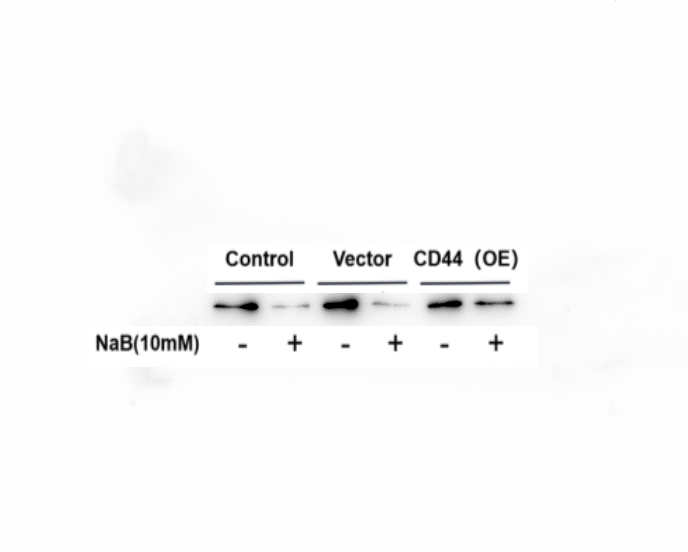

Supplement: Supplementary file 1 [file cancers-15-00423-s001.zip › supplementary figures/Figure S3. The original Western blotting figures of Figure 5B/slc7a11/3.png]

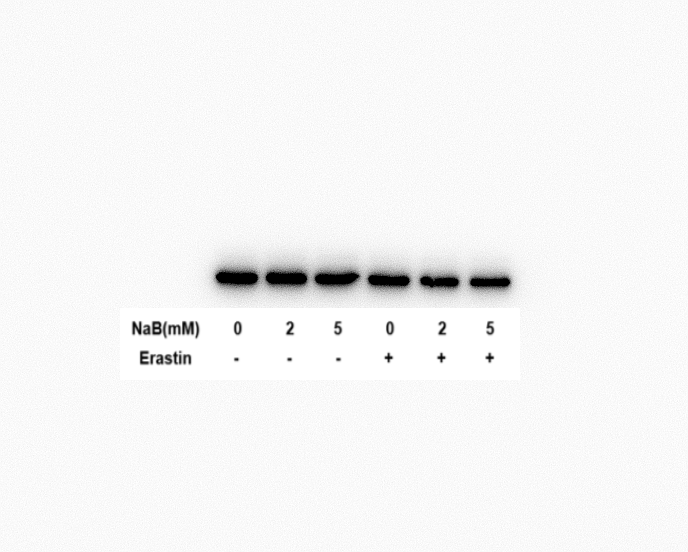

Supplement: Supplementary file 1 [file cancers-15-00423-s001.zip › supplementary figures/Figure S4. The original Western blotting figures of Figure 6G/gapdh/1.png]

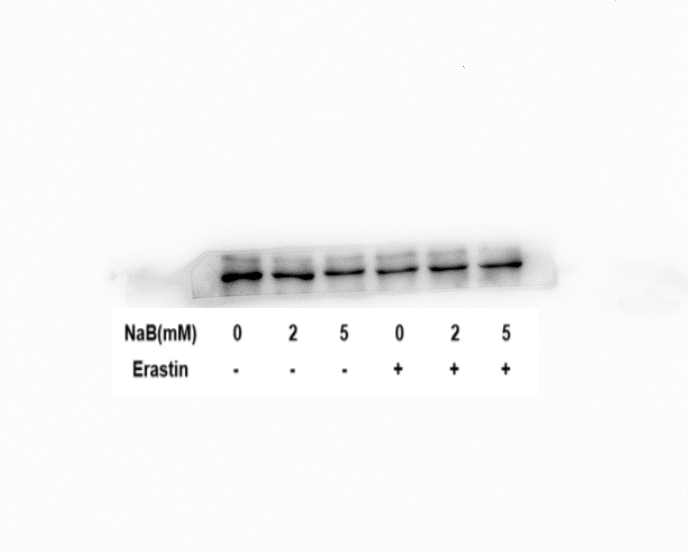

Supplement: Supplementary file 1 [file cancers-15-00423-s001.zip › supplementary figures/Figure S4. The original Western blotting figures of Figure 6G/gapdh/2.png]

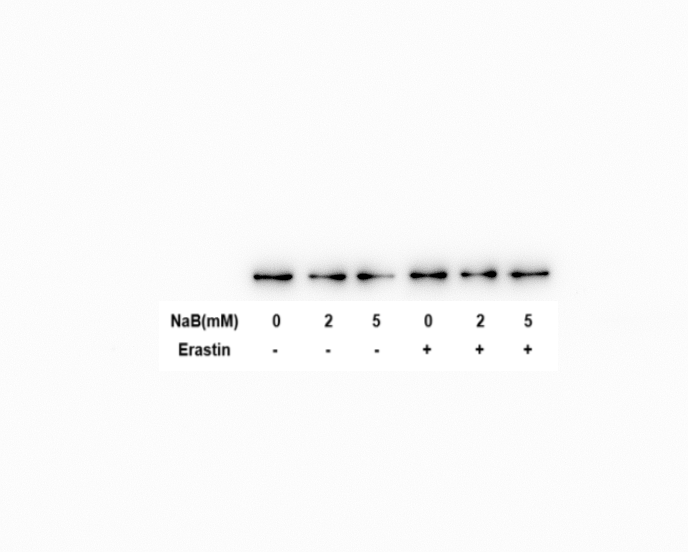

Supplement: Supplementary file 1 [file cancers-15-00423-s001.zip › supplementary figures/Figure S4. The original Western blotting figures of Figure 6G/gapdh/3.png]

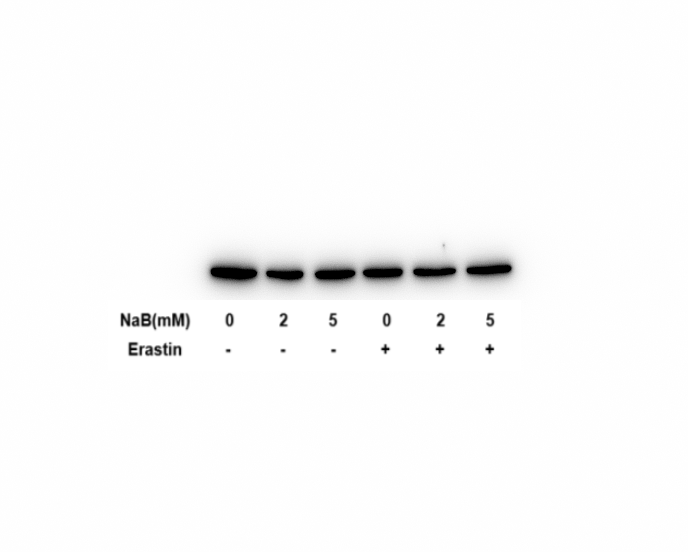

Supplement: Supplementary file 1 [file cancers-15-00423-s001.zip › supplementary figures/Figure S4. The original Western blotting figures of Figure 6G/gapdh/4.png]

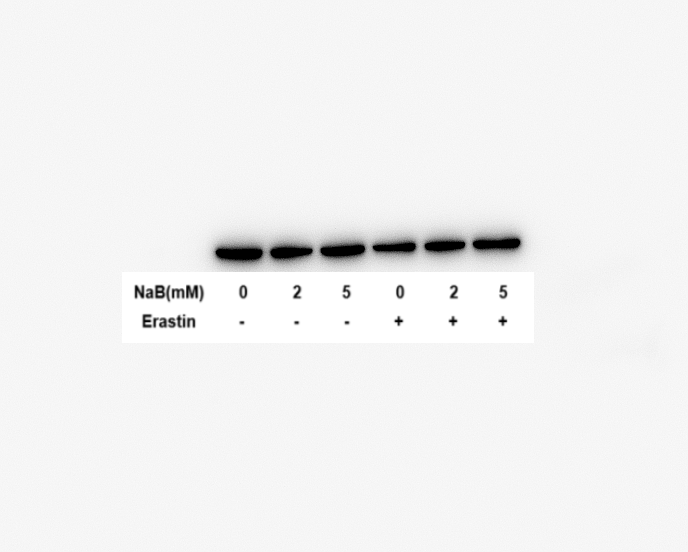

Supplement: Supplementary file 1 [file cancers-15-00423-s001.zip › supplementary figures/Figure S4. The original Western blotting figures of Figure 6G/gapdh/5.png]

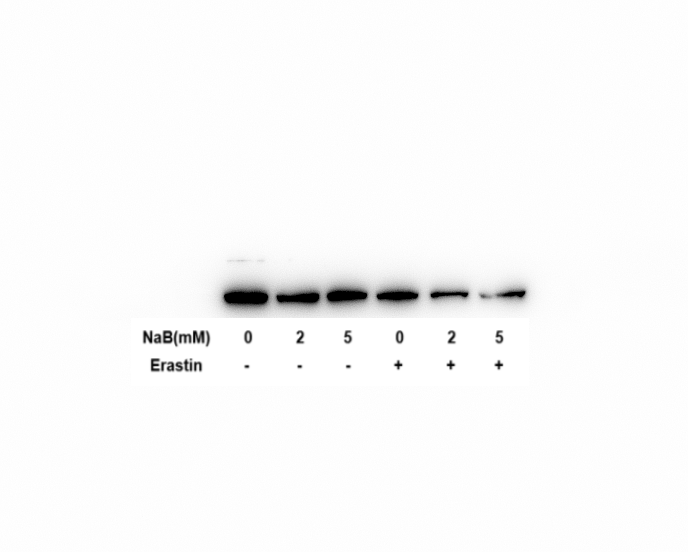

Supplement: Supplementary file 1 [file cancers-15-00423-s001.zip › supplementary figures/Figure S4. The original Western blotting figures of Figure 6G/slc7a11/1.png]

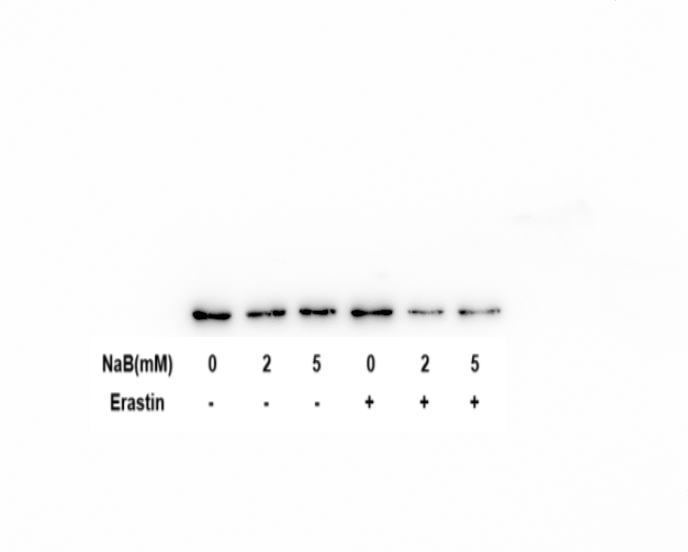

Supplement: Supplementary file 1 [file cancers-15-00423-s001.zip › supplementary figures/Figure S4. The original Western blotting figures of Figure 6G/slc7a11/2.png]

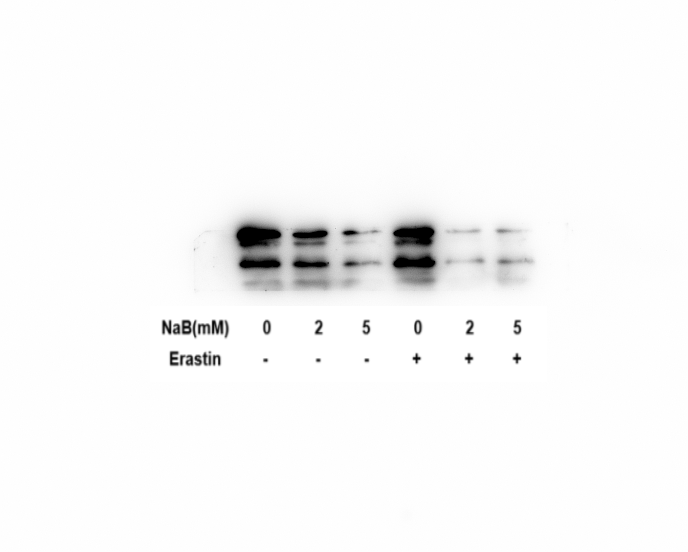

Supplement: Supplementary file 1 [file cancers-15-00423-s001.zip › supplementary figures/Figure S4. The original Western blotting figures of Figure 6G/slc7a11/3.png]

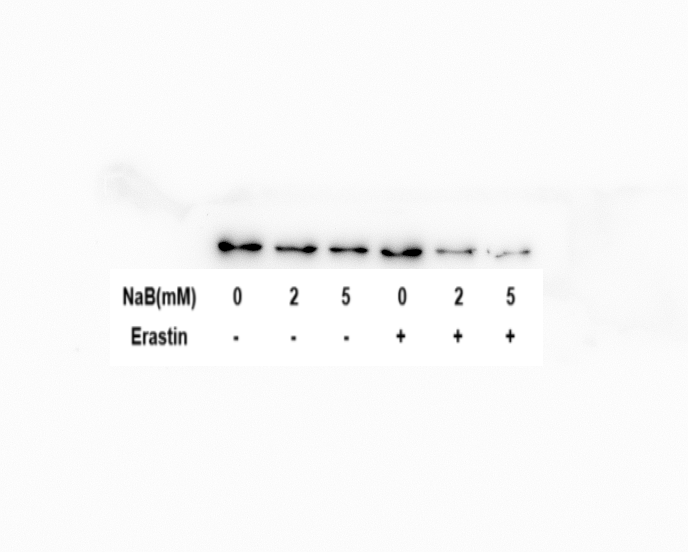

Supplement: Supplementary file 1 [file cancers-15-00423-s001.zip › supplementary figures/Figure S4. The original Western blotting figures of Figure 6G/slc7a11/4.png]

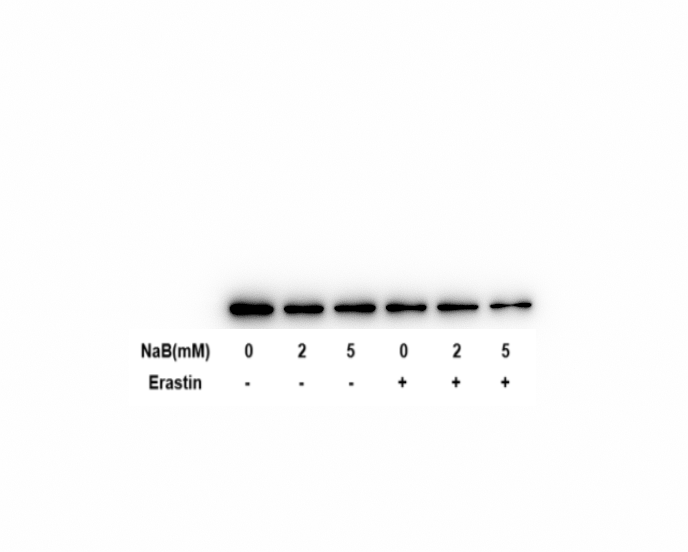

Supplement: Supplementary file 1 [file cancers-15-00423-s001.zip › supplementary figures/Figure S4. The original Western blotting figures of Figure 6G/slc7a11/5.png]
